# Diagnosing Trauma-related Dissociative Disorders in Hungary: The Development of the Hungarian Version of MID (MID-HU)

**DOI:** 10.1192/j.eurpsy.2024.769

**Published:** 2024-08-27

**Authors:** Z. Boytha, Á. Münnich, J. Molnár

**Affiliations:** ^1^Institute of Behavoural Sciences, University of Debrecen, Debrecen, Hungary

## Abstract

**Introduction:**

The recognition, diagnostics and treatment of dissociative disorders (DD) in Hungary is currently in its infancy. According to international researches the prevalence of dissociative disorders is similar to that of the major psychiatric disorders (bipolar disorder, schizophrenia, etc.). Due to the lack of valid diagnostic tools no data is available regarding the prevalence of dissociative disorders in Hungary so far.

**Objectives:**

To fill this gap within our profession; to provide a complex diagnostic tool; developing the hungarian version of the Multidimensional Dissociation Questionnaire (MID-HU)

**Methods:**

341 people participated in our study classified into four groups: (1) healthy controls (n=88), (2) patients from private practice diagnosed with DD and all those participants who have DD according to their MID results (n=103), (3) hospitalized psychiatric (mixed sample, n=60) and (4) SUD patients (n=89). The questionnaire package contained the hungarian version of the Multidimensional Inventory of Dissociation (MID-HU), the Dissociative Experience Scale (DES), the Traumatic Antecendents Questionnaire (TAQ), the Self-Report Version of the Dissociative Disorders Interview Schedule (DDIS-SR) and additional questions. Now we present the first results regarding the adaptation process of the hungarian MID (MID-HU).

**Results:**

The mean age of the participants was 36 years, 61,6% were female and 38,4% male.

The MID-HU has strong internal consistency: the alpha coefficients for the 14 facet scales were 0.88 or higher. The alpha coefficients for the 23 dissociation diagnostic scales ranged from 0,74-0,95; 9 were excellent (0,90 or above), 10 were good (0,80 or above), and 4 were fair (0,70 or above). The test-retest correlation of the Mean MID-HU scores is good (0,87). Factor analysis of the MID-HU extracted one main factor: dissociation. The mean MID-HU scores correlated with mean DES scores (0,87), indicating a good convergent validity. We found significant differences between the healthy control group and the dissociatve group in the mean DES (control: 9,5, dissociative: 27,6, Sig:<0,001), mean MID (control: 2,5, dissociative: 27, Sig: <0,001), the 23 dissociative diagnostics scales of the MID, 14 facet scales of the MID, and all diagnostics scales of the DDIS (using Mann-Whitney).

**Conclusions:**

The MID-HU seems to be a valid instrument, that can differentiate between DD patients and healthy people.

**Disclosure of Interest:**

None Declared

